# Prevalence of the Acute Respiratory Infections and Associated Factors in the Rural Areas and Urban Slum Areas of Western Maharashtra, India: A Community-Based Cross-Sectional Study

**DOI:** 10.3389/fpubh.2021.723807

**Published:** 2021-10-26

**Authors:** Sujata Murarkar, Jayashree Gothankar, Prakash Doke, Girish Dhumale, Prasad D. Pore, Sanjay Lalwani, Sanjay Quraishi, Reshma S. Patil, Vivek Waghachavare, Randhir Dhobale, Kirti Rasote, Sonali Palkar, Nandini Malshe, Rupeshkumar Deshmukh

**Affiliations:** ^1^Medical College Pune, Bharati Vidyapeeth (Deemed to be) University, Pune, India; ^2^Medical College Sangli, Bharati Vidyapeeth (Deemed to be) University, Sangli, India; ^3^Department of Community Medicine, Symbiosis Medical College for Women, Symbiosis International University, Pune, India

**Keywords:** acute respiratory infections, under-five children, rural area, urban slums, parental smoking

## Abstract

Acute respiratory infections (ARIs) continue to be the most important cause of morbidity and mortality among under-five children. Some demographic and environmental factors are associated with ARIs among under-five children. This study was conducted with the objective to estimate the prevalence of ARIs among under-five children in the rural areas and densely populated urban slum areas in Maharashtra, India and to assess the association of the selected sociodemographic and household environmental factors with ARI. This study was conducted in 16 selected clusters from the rural areas and densely populated urban slum areas of the two districts in Maharashtra, India. Structured and validated proforma was used for collecting the data on the sociodemographic and household environmental risk factors. A total of 3,671 under-five children were surveyed. The prevalence of ARIs for the preceding month was 50.4%. It was higher among the children living in the rural areas (54.2%) compared to the children living in the urban areas (46.7%) (*p* = 0.01). The prevalence of ARIs was reported to be 51.4 and 49.4% in boys and girls, respectively. In the multivariate analysis, the researchers found that living in rural areas (*p* = 0.01) and parental smoking (*p* = 0.04) were significantly associated with the ARIs. An intervention such as reducing parental smoking habits at the household level may reduce ARIs.

## Introduction

Acute respiratory infection (ARI) is a disease of public health significance. It is caused by a heterogeneous group of organisms that affects the human airways (1). It affects all ages, but the effects are particularly life-threatening among under-five children (2).

Globally, ARIs (predominantly pneumonia) have a 20% of mortality among children <5 years old. If neonatal pneumonia is also considered, the mortality increases to 35–40% among under-five children, accounting for 2.04 million deaths/year. Southeast Asia has the highest incidence of ARI followed by the sub-Saharan African countries; together, they contribute to more than 80% of the total global cases (3). Multiple social and environmental factors affect the morbidity and mortality of ARI in childhood. Factors include poverty, poor nutrition, poor housing conditions, indoor air pollution (including parental smoking), poor ventilation, overcrowding, industrialization, sociocultural values, overuse and misuse of antibiotics, lack of basic health services, and lack of awareness (4). It is also important to note that a quarter of ARI deaths in children are attributable to passive smoking (5). The National Family Health Survey 5, conducted in 2019–2020, reported a 2.4% prevalence of ARI in the preceding 2 weeks in the urban areas and a 3.8% in the rural areas in Maharashtra state (6). In the Indian slum areas, ARI constitutes more than two-thirds of all childhood illnesses (7). Globally, in 2010, nearly 265,000 in-hospital deaths of young children were attributed to ARI, 99% of which were reported in developing countries (8). In the urban slum areas, ARI constitutes over two-thirds of all childhood illnesses (9). In India, 14.3% of the deaths among infants and 15.9% of the deaths among children between 1 and 5 years of age are due to ARIs and most of these deaths are preventable. Because of the high morbidity and mortality rates associated with ARIs, its control continues as a major challenge to the healthcare system (10).

Hence, a comprehensive understanding of the prevalence and associated factors of ARI in rural areas and urban slum areas is essential. The majority of the previous studies on ARI had been conducted either in rural areas or in urban areas. By inclusion of both the urban and rural areas, this study attempts to meet the gap. Therefore, the main objective of this study was to estimate the prevalence of ARIs among under-five children in the rural areas and densely populated urban slum areas in Maharashtra, India and to assess the association of the selected sociodemographic and household environmental factors with ARI.

## Materials and Methods

This was a community-based cross-sectional study conducted among the children aged <5 years in 16 clusters of the two districts of western Maharashtra, India. Here, cluster means a revenue village or densely populated urban area, and it is also known as an urban slum. In this study, eight trained field supervisors collected the data with the cooperation of the accredited social health activist (ASHA) and the Anganwadi workers (AWWs). A faculty from either the community medicine or pediatrics monitored the quality of the collected data.

This was a part of the baseline survey of a cluster randomized controlled trial. The trial was registered with the Clinical Trial Registry of India. The duration of the main study was from December 15, 2015, to March 14, 2018. Data collection of this study was done between February 15, 2016, and May 14, 2016.

### Selection of Clusters

**Figure 1:**
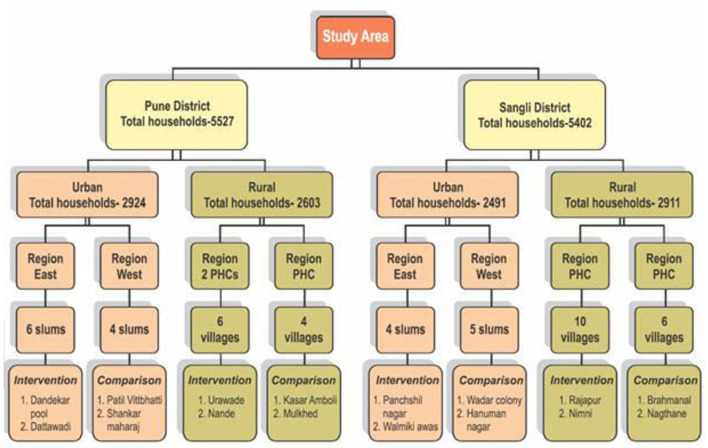
Study area and selection of clusters.

In both the districts, urban field practice areas were grouped into two, i.e., the East or West regions, and rural areas were grouped into the areas of two primary healthcare (PHC). From each region, two clusters each were randomly selected by using the random numbers generated by Microsoft Excel. Thus, a total of 16 clusters were selected. Figure 1 shows a flowchart of the selection of the clusters from the study area.

### Study Population and Sample Size

The estimated (based on the population and age group distribution 2011 census) average number of under-five children per cluster was 250. Considering the ARI prevalence of 27% (11) with a 95% CI and allowable difference of 10%, the sample size calculated by using the Z^2^α p (1-p)/d^2^ formula was 1,691. With a design effect of two, the sample size was estimated to be 3,382.

### Research Tool

The interview schedule was prepared in English. It was validated by the experts and then translated into the local language (Marathi) and retranslated to English. A unique identification code was given to each house, the mother of a child below 5 years and the child.

### Data Collection

Investigators had appointed the eight field supervisors (FSs) for carrying out the survey. Their work was monitored by the eight site investigators (SIs). We obtained written informed consent from the mother of under-five children. Field supervisors collected the data through house-to-house visits with the support of the ASHA and the AWWs. FSs were cross-checked the data collected by the SIs to ensure completeness and accuracy before the data entry.

All the households with at least one under-five children in the selected clusters were visited. All the eligible children from the particular household were enrolled. Information about the locked houses was solicited from the neighbors. These houses were visited again in the evening, in the early morning, or on the weekends. Locked houses during the second visit and the families who had permanently migrated from the area were excluded from this study. Information about the sociodemographic and household environmental factors such as overcrowding, the type of fuel used for cooking, parental smoking habits, and ventilation in the house was documented. Information about the total floor area of the house in square feet excluding the bathroom and water closet area and the number of doors and windows was obtained for the assessment of ventilation of the household. The supervisors asked the mothers regarding information related to ARIs in the preceding 1 month and recorded the responses from the mothers.

### Definition of Study Variables and Other Definitions Used in This Study

1. **Urban slum:** A compact area of at least 300 population or about 60–70 households with poorly built and congested tenements in the unhygienic environment usually with inadequate infrastructure and lacking the proper sanitary and drinking water facilities (12).
2. **Types of the family:** The family type was divided into only two groups; the nuclear family consists of the husband, wife, and unmarried children staying together and the joint family included all the other families including the three-generation family and extended family.
3. **The economic status of the family:** In the state of Maharashtra, under the Public Distribution System (PDS), cards of the three different colors are provided to the families according to their annual income as follows: families who earn up to Rs. 15,000 (US $205.97) receive a yellow ration card, families who earn between Rs. 15,000 to 1 lakh (the US $205.97–1373.15) receive an orange ration card, and families who earn more than Rs. 1 lakh (the US $1373.15) receive a white ration card (13). The color of the ration card was considered a proxy for income.
4. **Level of education of the mothers:** The level of education was classified according to the number of years of schooling. A person was considered illiterate if he/she was unable to read or write (14). According to the years of schooling, educational status was classified as education up to 6th standard, 7th−10th standard, 12th standard/Diploma, Graduate, and above.
5. **Exclusive breastfeeding:** Child fed only breast milk, except taking the vitamins, mineral supplements, or medicines, until 6 months of age, was considered as exclusive breastfeeding (15).
6. **Ventilation:** Inadequate ventilation was defined as <50 square feet of floor space area per person and the absence of the doors and windows facing each other (16).
7. **Overcrowding:** Accepted standards of the number of per person per room were used. If the number of persons per room is more than these criteria, overcrowding was considered to be existing (16).
8. **Types of fuel:** The fuel used in a household was classified as either clean or unclean by considering the most common fuel used for cooking. Clean fuels included liquefied petroleum gas (LPG) or electricity and unclean fuels included biomass, coal, and kerosene (14).
9. **Acute respiratory infection:** ARI was defined as an episode of coughing accompanied by nasal discharge and/or shallow, rapid breathing, and/or difficulty in breathing (17) in the month preceding the survey as reported by the mother of the child.
10. **Accredited social health activist:** The ASHA works at the village level under the National Health Mission program of India. She creates awareness of the health in the community (16).
11. **Anganwadi worker:** Under the Integrated Child Development Services (ICDS) scheme, the AWWs (part-time workers) are appointed to render the health services in the community (16).

### Data Management

All the filled forms were entered into a software database. Critical fields in the tool were identified to check the completeness and accuracy of the form. All the critical fields and few non-critical fields were monitored. Discrepancies up to 0.1% for the critical data and up to 1% for the non-critical data were considered acceptable. For the discrepancies related to data entry, the alternate forms were physically cross-checked. Statisticians cleaned and analyzed the data by excluding the missing data.

### Data Analysis

Data were analyzed by using the Statistical Package for the Social Sciences (SPSS) (IBM SPSS Chicago USA version 25). Descriptive statistics (mean and SD) were calculated for the continuous variables and the frequencies and percentages were calculated to summarize the qualitative variables. The multivariate logistic regression analysis was carried out to identify the determinants of ARI. *p* < 0.05 was considered as statistically significant.

## Results

A total of 3,671 under-five children were included in this study. There were 1,834 under-five children in the urban slum areas and 1,837 under-five children in the rural areas. There were 1,732 girls and 1,939 boys in the study areas. The mean age of the children was 2.38 years (±SD 1.36) and the mean age of the mothers was 24.25 years (±SD 6.37).

### Housing Environment

Overcrowding has existed in 43% of the households. A majority, i.e., 94.4% of the households were inadequately ventilated. A higher proportion of households in urban clusters was inadequately ventilated compared to the rural clusters (*p* < 0.05). The prevalence of indoor smoking was 3.2 and 2.1% in the urban and rural clusters, respectively (*p* < 0.05). Out of the total households, 15.1% used unclean fuel. The use of unclean fuel is higher in the rural clusters (24.0%) compared to the urban clusters (6.0%).

The children with ARIs stratified by the sociodemographic factors are described in Table 1. The overall prevalence of ARIs in the study was 50.4%. It was higher among the children living in the rural areas (54.2%) compared to the children living in the urban areas (46.7%) (*p* = 0.01). Most of the mothers were literate and their educational status was not associated with the prevalence of ARI.

**Table 1:** Distribution of the children with acute respiratory infection (ARI) according to the sociodemographic factors.

**Variables**	**ARIs (***N*** = 1,852) (%)**	**No ARIs (***N*** = 1,819) (%)**	***P*** **-value**	**Adjusted odds ratio 95%CI**
**Age group (months)**				
≤ 23 months	809 (53.22)	711 (46.78)		1
>23 months	1,043 (48.49)	1,108 (51.51)	0.99	NA*
**Sex of children**				
Male	997 (51.42)	942 (48.58)		1
Female	855 (49.36)	877 (50.64)	0.77	0.94 (0.62–1.42)
**Type of family**				
Nuclear	763 (49.97)	764 (50.03)		1
Joint	1,081 (50.80)	1,047 (49.20)	0.62	0.89 (0.55–1.42)
**Income of family**				
≤1373.15 USD	1,033 (48.89)	1,080 (51.11)		1
>1373.15 USD	790 (52.70)	709 (47.30)	0.072	1.51 (0.96–2.36)
**Place of residence**				
Rural	996 (54.22)	841 (45.78)		1
Urban	856 (46.67)	978 (53.33)	0.005	0.50 (0.31–0.82)
**Exclusive Breastfeeding**				
Yes	54 (46.15)	63 (53.85)		1
No	187 (58.07)	135 (41.93)	0.62	1.14 (0.68–1.90)
**Maternal education**				
≤6th Std	257 (47.24)	287 (52.76)		1
>6th Std	1,538 (51.03)	1,476 (48.97)	0.14	0.63 (0.34–1.16)

* *Range is from 0 to almost infinity*.

Most of the houses in the study area were overcrowded and the majority of the households used clean fuels for cooking. Most of the children who had ARIs were exposed to parental smoke (Table 2).

**Table 2:** Distribution of the children with ARI according to the household environmental factors.

**Variables**	**ARIs (***n*** = 1,852) (%)**	**No ARIs (***n*** = 1,819) (%)**	***P*** **-value**	**Adj. odds ratio 95%CI**
**Type of fuel**				
Clean	1,553 (49.79)	1,566 (50.21)		1
Unclean	291 (54.29)	245 (45.71)	0.88	1.05 (0.53–2.08)
**Overcrowding**				
Yes	1,226 (49.12)	1,270 (50.88)	0.08	1
No	617 (53.42)	538 (46.58)		1.56 (0.95–2.56)
**Ventilation**				
Adequate	64 (52.03)	59 (47.97)		1
Inadequate	1,719 (50.60)	1,678 (49.40)	0.006	0.12 (0.03–0.54)
**Parental smoking**				
Yes	73 (55.73)	58 (44.27)		1
No	1,769 (50.27)	1,750 (49.73)	0.035	0.32 (0.11–0.92)

In the multivariate logistic regression analysis, researchers found that residence in the rural areas (*p* = 0.01) and parental smoking (*p* = 0.04) were significantly associated with the higher prevalence of ARIs. Strangely inadequate ventilation did not have a negative association (*p* = 0.01).

## Discussion

It is reported that Bangladesh, India, Indonesia, and Nepal together account for 40% of the global mortality of ARI (18). ARI is the third most common individual cause of death in both developed and developing countries (1). The prevalence of ARI reported by the various studies ranges from 20 to 30% (7, 19, 20). This study found the prevalence of ARI to be 50.4% among under-five children which is similar to the other studies (6, 11). A study was done in Karnataka (a state in India) noted an ARI prevalence of <10% in under-five children (18). Based on the differences in the socioeconomic, cultural, and environmental factors present in the different geographical regions, the prevalence of ARI varies. Some studies have reported a higher prevalence of ARIs in the rural areas compared to the urban areas as observed in this study (4, 11). In this study, inadequate ventilation did not lead to higher ARI. There may be several reasons, such as pets in the family and history of ARI in the family members, which the authors did not substantiate. A few studies reported that the children living in homes with poor ventilation in the rural areas developed more ARIs compared to the children living in the homes with poor ventilation in the urban areas (1, 4, 7, 19). Under-five children living in houses with inadequate ventilation contract more ARIs compared to under-five children living in well-ventilated houses because a lack of ventilation implies that indoor smoke is trapped and the toxic components accumulate in the houses and affect the respiratory systems of the children, leading to the development of ARIs (21). The burning of unclean fuel such as dung, wood, crop residues, and coal leads to the accumulation of smoke in houses with inadequate ventilation. This smoke has a powerful effect on the lungs of under-five children, who spend a substantial amount of time indoors, leading to an increase in the risk of developing repeated ARIs. In India, the government launched Pradhan Mantri Ujjwala Yojana (PMUY) in 2016, providing free LPG connections to the below poverty line (BPL) families. Successful implementation of such schemes will empower the women and protect their health and the health of their children (22). In this study, the use of unclean fuel for cooking was more common in rural areas compared to the urban areas, but the use of unclean fuel was not found to be a significant contributor to ARIs in under-five children. Another important household environmental factor responsible for ARI among under-five children is parental smoking habits. The prevalence of ARI is usually higher among the children from the rural areas with a history of parental smoking (4, 11, 23) compared to the children from the densely populated urban areas with a history of parental smoking (7, 24). The effect of smoke on the prevalence of ARI might be enhanced by inadequate ventilation (25).

This study showed an equal prevalence of ARIs among boys and girls, which was similar to a study performed in Eastern Indonesia in the urban areas (26). However, other studies have reported a greater prevalence of ARIs in boys compared to girls (1, 10). Only one study (7) found that girls were more prone to ARIs than boys.

Generally, education helps to improve the knowledge of maternal caretaking with respect to the risk factors responsible for ARIs, but this study revealed no difference in the prevalence of ARIs in the children born to mothers with varied educational levels. This was in contrast to the results of many studies (1, 10, 17, 19), which have shown a higher prevalence of ARIs in the children born to mothers with primary education levels compared to those born to highly educated mothers.

### Strengths and Limitations

This study had several strengths. First, this community-based multisite study was conducted among a vulnerable group of children from the rural and densely populated urban areas of India. Information regarding the household environment was not self-reported by the head of the household but rather it was collected and confirmed during the house-to-house survey. Second, support of the ASHA and the AWWs (health workers of the government sector) was sought in the data collection. The institute of the authors is a private medical college that represents the public-private relationship.

Seasonal variations could not be captured in this study, as it was performed over a shorter duration that would be necessary to investigate the seasonal swings in the occurrence of ARI.

## Conclusion

The prevalence of ARI was higher in the children living in the rural areas and among under-five children with parental smoking habits. An intervention, such as reducing the parental smoking habits at the household level may reduce ARIs.

## Data Availability Statement

The raw data supporting the conclusions of this article will be made available by the authors, without undue reservation.

## Ethics Statement

The studies involving human participants were reviewed and approved by Bharati Vidyapeeth Deemed University, Medical College Pune, Institutional Ethics Committee (ECR/313/Inst/MH/2013/RR-16) and Department of Pharmacology Bharati Vidyapeeth Deemed University Medical College and Hospital Sangli (ECR/276/Inst/MH/2013/RR-16). Written informed consent to participate in this study was provided by the participants' legal guardian/next of kin.

## Author Contributions

JG, PD, PP, GD, and SL contributed to the framing of the design of the study. SQ, SM, RP, SP, VW, RDh, KR, and NM were responsible for the data collection. JG, PP, GD, and PD analyzed and interpreted the data with inputs from SL, SQ, SM, RP, NM, SP, VW, RDe, and KR. SM has drafted the manuscript. All authors were involved in revising the manuscript critically.

## Funding

This work was supported by the Bill and Melinda Gates Foundation (OPP1084307) through the INCLEN Trust International, New Delhi, India (Project ID: INC2015GNT006). The Bill and Melinda Gates Foundation and the INCLEN Trust International had no role in the design of the study, data collection, data analysis, data interpretation, or writing of the manuscript.

## Conflict of Interest

The authors declare that the research was conducted in the absence of any commercial or financial relationships that could be construed as a potential conflict of interest.

## Publisher's Note

All claims expressed in this article are solely those of the authors and do not necessarily represent those of their affiliated organizations, or those of the publisher, the editors and the reviewers. Any product that may be evaluated in this article, or claim that may be made by its manufacturer, is not guaranteed or endorsed by the publisher.
