# Supplementary material for: Prevalence of the Acute Respiratory Infections and Associated Factors in the Rural Areas and Urban Slum Areas of Western Maharashtra, India: A Community-Based Cross-Sectional Study
Source: Front Public Health. 2021 Oct 26;9:723807. doi: 10.3389/fpubh.2021.723807 (PMC8576147; doi:10.3389/fpubh.2021.723807)
Supplement: Supplementary file 2 [file Table_1.DOCX]

STROBE Statement—Checklist of items that should be included in reports of ***cross-sectional studies***

# Item

|  | **No** | **Recommendation** |
| --- | --- | --- |
| **Title and abstract** | 1 | (*a*) Prevalence of Acute respiratory infections (ARIs) and associated factors in the rural area and urban slums of western Maharashtra, India: a cross-sectional community-based study. |
|  |  | (*b*) Background Acute respiratory infections (ARIs) continue to be the most important cause of morbidity and mortality among under-five children. Some demographic and environmental factors are associated with ARIs among under-five children. Present study was conducted with the objectives to estimate the prevalence of ARIs among under-five children in rural areas and densely populated urban slums in Maharashtra, India, and to assess association of the selected sociodemographic and household environmental factors with ARI. Study was conducted in 16 selected clusters from rural areas and densely populated urban areas of two districts in Maharashtra, India. Structured and validated proforma was used for collecting data on sociodemographic and household environmental risk factors. Total 3,671 under-five children were surveyed. The prevalence of ARIs for the preceding one month was 50.4%. It was higher among children living in rural areas (54.2%) than children living in urban areas (46.7%) (p =0.01). Prevalence of ARIs was reported to be 51.4% and 49.4% in boys and girls respectively. In Multivariate analysis, the researchers found that living in a rural area (p= 0.01) and parental smoking (p=0.04) were significantly associated with the ARIs. Intervention like reducing parental smoking habits at the household level may reduce ARIs. |
| **Introduction** |  |  |
| Background/rationale | 2 | Globally, acute respiratory tract infections (ARIs) (predominantly pneumonia) have a 20% mortality among children less than 5 years old. If neonatal pneumonia is also considered, the mortality is approximately 35-40% among under-five children, accounting for 2.04 million deaths/year. Southeast Asia has the highest ARI incidence, accounting for more than 80% of the total global cases, together with sub-Saharan African countries. It is reported that Bangladesh, India, Indonesia and Nepal together account for 40% of the global ARI mortality. The ARI mortality rates among children are ten to fifty times greater in developing countries than in developed countries. ARI is the third most common individual cause of death in both developed and developing countries. There are multiple social and environmental factors associated with ARI morbidity and mortality in childhood like poverty, poor nutrition, poor housing conditions, indoor air pollution such as parental smoking, absence of ventilation, overcrowding, industrialization, social cultural values, overuse and misuse of antibiotics, lack of basic health services and lack of awareness. Risk of respiratory infections increases when children are exposed to second-hand smoke. It is also important to consider that a quarter of ARI deaths in children are attributable to passive smoking. Burden of ARI in the community is underestimated. Hence, continued understanding of ARI prevalence and associated risk factors in rural area and urban slum is essential. Majority of the previous studies on ARI had been conducted either in rural area or in the urban area. This study is aimed at addressing this gap. |
| Objectives | 3 | 1. To estimate prevalence of Acute respiratory infections (ARIs) among under five children 2. To assess association of the selected sociodemographic and household environmental factors with ARI. |
| **Methods** |  |  |
| Study design | 4 | A community- based cross- sectional study |
| Setting | 5 | Current study was carried out in rural areas and urban slums in two districts in Maharashtra state, India. The rural and urban areas have revenue villages and slums respectively. These slums or revenue villages are referred as clusters. There are total 45 clusters (Pune district =20, Sangli district = 25). On an average there are 250 under five children per cluster and total population of 1, 89,504.  periods of recruitment and training of manpower- 15th December 2015 to 14th February 2016  dates of data collection- 15 Feb 2016 to 14 May 2016. |
| Participants | 6 | (*a*) All children below five years with their mothers in the selected areas. In the selected clusters, families with under-five children residing for more than six months were included in the study. Households which were found to be locked during two consecutive visits were not included in the study. |
| Variables | 7 | 1. Type of family: Family type was divided into only two groups; the nuclear family consists of the husband, wife and unmarried children staying together, the joint family included all other families including three generation family as well as extended family.  2. Economic status of the family:  In the state of Maharashtra, under the Public Distribution System (PDS), cards of three different colors are provided to families according to their annual income as follows: families who earn up to Rs. 15,000 (US $205.97) receive a yellow ration card, families who earn between Rs. 15,000 to 1 lakh (US $205.97 to 1373.15) receive an orange ration card, and families who earn more than Rs. 1 lakh (US $1373.15) receive a white ration card. Color of ration card was considered proxy for income.  3. Level of education of mothers:  The level of education was classified according to the number of years of schooling. A person was considered illiterate if he was unable to read or write. According to years of schooling educational status was classified as education up-to 6th standard, 7th to 10th standard, 12th standard/Diploma, Graduate and above.  4. Exclusive breastfeeding: Child fed only breast milk except taking vitamins, mineral supplements, or medicines until six months of age was considered as exclusive breastfeeding.  5. Ventilation: Inadequate ventilation was defined as less than 50 square feet of floor space area per person and absence of doors and windows facing each other.  6. Overcrowding: Accepted standards of number of per person per room were used. If the number of persons per room is more than these criteria, overcrowding was considered to be existing.  7. Type of fuel: The fuel used in a household was classified as either clean or unclean by considering the most common fuel used for cooking. Clean fuels included liquefied petroleum gas (LPG) or electricity, and unclean fuel included biomass, coal, and kerosene.  8. ARI: ARI (Acute respiratory infection) was defined as an episode of coughing accompanied by nasal discharge and/or shallow, rapid breathing and/or difficulty in breathing in the month preceding the survey, as reported by the child’s mother. |
| Data sources/ measurement | 8* | History of ARI (Last one month) preceding survey was asked to mothers of under-five children. The exact age of the child was computed from the child’s date of birth. When data on exact date of birth was not available, the age as told by mother was used, to nearest month. |
| Bias | 9 | Efforts were taken in to minimize the selection bias by random selection of the study clusters. Adequate training in data collection, validation of the study tool and daily calibration of the study instruments, use of standardized techniques ensured reduction in biases. |
| Study size | 10 | Sample size was determined using an estimated prevalence of 27% for ARIs .With a 95% confidence interval and allowable difference of 10% of prevalence, sample size 1691 was calculated by using the following formula.  Sample size = Z2α p (1-p)/d2  With design effect 2, the sample size came around 3382, but we had included 3671 children which is an adequate sample size. |
| Quantitative variables | 11 | Quantitative variable findings were shown by descriptive statistics |
| Statistical methods | 12 | (*a*) Data were analyzed using statistical package for social sciences (SPSS) (version 20). Descriptive statistics (mean and standard deviation) were calculated for continuous variables and frequencies and percentages were calculated to summarize qualitative data. Multivariate logistic regression analysis was carried out to identify the determinants of acute respiratory infection (ARI). P < 0.05 was considered as significant. |
|  |  | (*b*) Describe any methods used to examine subgroups and interactions |
|  |  | (*c*) Explain how missing data were addressed-the data was excluded |
|  |  | (*d*) If applicable, describe analytical methods taking account of sampling strategy |
|  |  | (*e*) Describe any sensitivity analyses |
| **Results** |  |  |
| Participants | 13* | (a) Total 3,671 under five children were covered from rural area and urban slum. Mean age of the children was 2.38 years (± SD 1.365). |
|  |  | b) Give reasons for non-participation at each stage- For 129 children, immunization information was missing as their status could not be confirmed by immunization card. Hence not included in the study |
|  |  | (c) Consider use of a flow diagram |
| Descriptive data | 14* | (a) Give characteristics of study participants (eg demographic, clinical, and social) and information on exposures and potential confounders- In the present study there were 2,929 mothers with 3,671 under five children.. There were 1,834 under-five children in urban slum and 1,837 were in rural area. There were 1,939 boys and 1,732 girls in the study area. Mean age of the children was 2.38years (±SD 1.365). Out of these 3,671 under five children, 752 were infants. Mean birth weight of these infants was 2.6kg ±0.61. Mean age of mothers was 24.25±6.37 years. There were total 768 children in the age group of 12 to 23 months but only 639 children possessed immunization card. |
|  |  | (b) Indicate number of participants with missing data for each variable of interest- There were total 768 children in the age group of 12 to 23 months but only 639 children possessed immunization card. For 129 children, immunization information was missing as their status could not be confirmed by immunization card. |
| Outcome data | 15* | Report numbers of outcome events or summary measures-There was one outcome variable i.e. Acute respiratory infections (ARIs). The overall prevalence of ARIs in the study was 50.4%, it was higher among children living in rural areas (54.2%) than among children living in densely populated urban areas (46.7%). |
| Main results | 16 | (*a*) The overall prevalence of ARIs in the study was 50.4%. It was higher among children living in rural areas (54.2%) than among children living in densely populated urban areas (46.7) (p =0.005). Most of the mothers were literate, and their educational status was not associated with the prevalence of ARI.  Most of the houses in the study area were overcrowded and the majority of the households used clean fuels for cooking. Most of the children who had ARIs were exposed to parental smoke (Table 2).  In Multivariate analysis researchers found that residence in a rural area(p=0.01), and parental smoking (P= 0.04). were significantly associated with the higher prevalence of ARIs. Strangely inadequate ventilation did not have negative association (p= 0.01). |
|  |  |  |
|  |  |  |
| Other analyses | 17 | Not applicable |

**Discussion**

Key results It is reported that Bangladesh, India, Indonesia and Nepal together account for 40% of the global ARI mortality(^[[1]](#endnote-1)^). ARI is the third most common individual cause of death in both developed and developing countries(^[[2]](#endnote-2)^). The ARI prevalence reported by various studies ranges from 20 to30% (7, ^[[3]](#endnote-3)^, ^[[4]](#endnote-4)^). Present study found 50.4% prevalence of ARIs among under-five children which is similar to other studies(6,11). A study done in Karnataka (state in India) noted an ARI prevalence of less than 10% in under-five children (18). Based on differences in socioeconomic, cultural and environmental factors present in different geographical regions, the prevalence of ARI varies. Some studies have reported a higher prevalence of ARIs in rural areas than urban areas, as observed in the current study (4,11). In the present study inadequate ventilation did not lead to higher ARI. There may be several reasons like pets in the family, history of ARI in family members etc. which authors did not substantiate. A few studies reported that children living in homes with poor ventilation in rural areas developed more ARIs than those living in homes with poor ventilation in urban areas (4, 7, 19, 20).Under-five children living in houses with inadequate ventilation contracts more ARIs than those living in well-ventilated houses, partly because a lack of ventilation implies that indoor smoke is trapped, and the toxic components accumulate in the houses and affect the respiratory systems of the children, leading to the development of ARIs(^[[5]](#endnote-5)^).The burning of unclean fuel such as dung, wood, crop residues and coal leads to the accumulation of smoke in houses with inadequate ventilation. This smoke has a powerful effect on the lungs of under-five children, who spend a substantial amount of time indoors, leading to an increase in the risk of developing repeated ARIs. In India, the government launched Pradhan Mantri Ujjwala Yojana (PMUY) in 2016, providing free LPG connections to below poverty line (BPL) families. Successful implementation of such schemes will empower women and protect their health as well as the health of their children(^[[6]](#endnote-6)^). In the current study, the use of unclean fuel for cooking was more common in rural areas than in urban areas, but the use of unclean fuel was not found to be a significant contributor to ARIs in under-five children. Another important household environmental factor responsible for ARI among under-five children is parental smoking habits. The prevalence of ARI is usually higher among children from rural settings with a history of parental smoking (4,11,^[[7]](#endnote-7)^) than among children from densely populated urban areas with a history of parental smoking(7 ,^[[8]](#endnote-8)^). The effect of smoke on the prevalence of ARI might be enhanced by inadequate ventilation (^[[9]](#endnote-9)^).

The present study showed an equal prevalence of ARIs among boys and girls, which was similar to a study performed in Eastern Indonesia in an urban setting(^[[10]](#endnote-10)^).However, other studies have reported a greater prevalence of ARIs in boys than in girls(10,19). Only one study (7) found that girls were more prone to ARIs than boys.

Generally, education helps improve maternal caretaking knowledge with respect to the risk factors responsible for ARIs, but our study revealed no difference in the prevalence of ARIs in children born to mothers with varied educational levels. This was in contrast to the results of many studies (10 ,17 ,19, 20), which have shown a higher prevalence of ARIs in children born to mothers with primary education levels compared to those born to highly educated mothers.

21 **Generalisability** The findings of the study can be generalized in the similar community based settings.

# Other information

Funding 22 Bill and Melinda Gates Foundation (OPP 1084307) through The INCLEN Trust International, New-Delhi, India (Project ID; INC2015GNT006).Apart from funding INCLEN trust international had given technical guidance for the present study.

*Give information separately for exposed and unexposed groups.

1. Taksande AM, Yeole M. Risk factors of acute respiratory infection (ARI) in under-fives in a rural hospital of central India. Journal of Pediatric and Neonatal Individualized Medicine. 2015; 5: 050105. [↑](#endnote-ref-1)
2. Akinyemi JO, Morakinyo OM. Household environment and symptoms of childhood acute respiratory tract infections in Nigeria, 2003-2013: A decade of progress and stagnation. BMC Infect Dis. 2018; 18(1):1–12. [↑](#endnote-ref-2)
3. Mir AA, Imtiyaz A, Fazili A, Iqbal J, Jabeen R, Salathia A. Prevalence and risk factor analysis of acute respiratory tract infections in rural areas of Kashmir valley under 5 years of age. Int J Med Public Health. 2012; 2: 47-52. [↑](#endnote-ref-3)
4. Prajapati B, Talsania N, Sonaliya K. A study on prevalence of acute respiratory tract infections (ARI) in under five children in urban and rural communities of Ahmedabad district, Gujarat. Natl J Community Med. 2011; 2: 255-259. [↑](#endnote-ref-4)
5. Gothankar J, Doke P, Dhumale G, Pore P, Lalwani S, Quraishi S, et al. Reported incidence and risk factors of childhood pneumonia in India: a community-based cross-sectional study. BMC Public Health. 2018;18: 1111. [↑](#endnote-ref-5)
6. Ministry of Petroleum and Natural Gas Governmnet of India Available from www.pmujjwalayojana.com/about.html accessed on 4/3/2018 [↑](#endnote-ref-6)
7. Tin ST, Woodward A, Saraf R, Berry S, Carr PA, Morton SMB, et al. Internal living environment and respiratory disease in children: findings from the growing up in New Zealand longitudinal child cohort study. Environ Health. 2016; 15: 120. [↑](#endnote-ref-7)
8. Lakshmi NB. Prevalence and factors associated with acute respiratory infections in children under five in urban slums of Bangalore city, Karnataka. Thiruvananthapuram, Kerala: Achutha Menon Centre for Health Science Studies; 2010. [↑](#endnote-ref-8)
9. Ujunwa F, Ezeonu C. Risk factors for acute respiratory tract infections in under-five children in Enugu Southeast Nigeria. Ann Med Health Sci Res. 2014; 4: 95-99. [↑](#endnote-ref-9)
10. Shibata T, Wilson JL, Watson LM, LeDuc A, Meng C, Ansariadi, et al. Childhood acute respiratory infections and household environment in an Eastern Indonesian urban setting. Int J Environ Res Public Health. 2014; 11: 12190-12203. [↑](#endnote-ref-10)
